# Note on a Camphor and Chloroform Mixture

**Published:** 1849-01

**Authors:** T. Smith, H. Smith

**Affiliations:** Chemist, Edinburgh; Chemist, Edinburgh


*Note on a Camphor and Chloroform Mixture.* By T. and H. Smith, Chemists, Edinburgh.—The great difficulty, or rather the utter impossibility, of administering camphor in a state of solution in doses of sufficient potency, has been long felt by the profession to be a serious evil. The form of pill, which forces itself on the physician as the only mode of giving large doses of this medicine, is objectionable in many cases, and in others altogether inadmissible. The camphor, being merely in a state of mechanical division, on being set free in the stomach, from its extreme lightness quickly separates and floats about, thus producing in many cases much local irritation in that organ, instead of soothing or arousing the general system.

We have, therefore, much pleasure in laying before the readers of the *Monthly Journal* a formula for exhibiting camphor in doses of almost any amount of strength—certainly as large as any case can require—and that in a state of perfect solution: thereby allowing of a nice adaptation of the dose to the circumstances of each case.

The formula is as follows:—Three drachms of solid camphor are dissolved in one fluid drachm of chloroform. This is, perhaps, one of the most remarkable cases of solution the whole range of chemistry presents to us. The solution is most *rapid and complete*, and the bulk of the liquid is now increased from one to fully four fluid drachms. This solution, rubbed up with the *yolk* of one fresh egg, may be formed into an extremely elegant emulsion by the addition of water, without the slightest separation of the camphor or chloroform; in fact, no separation of any kind takes place. If to the proportions given above, as much water be added as to make a four-ounce mixture, each teaspoonful of the mixture when formed will contain about five and a half grains of camphor, and about two minims of chloroform. The capability of the formula being varied, so that either the camphor or chloroform may constitute the predominating ingredient, must be quite obvious. This mixture can be administered in any ordinary vehicle, such as water, without the occurrence of any separation; indeed, the mixture is as readily and completely effected as cream with tea or coffee. We have tried the effect of several medicinal substances on the mixture. With none of them has any separation been caused.

A weak saline solution, composed of common salt, phosphate of soda, and an alkaline carbonate, mixed readily, as well as a solution of muriate of morphia and sulphate of zinc. With the volatile alkali and acid liquids—such as a weak solution of acetic and muriatic acids—the mixture seems to become more intimate and stable. The mixture with ammonia has stood since its preparation—now fully a week—without any separation. With water alone, however, the chloroform solution of camphor separates in a few days, but they readily unite again when slightly agitated. The solution of camphor in chloroform, although insoluble in water alone, appears in this mixture to be in as complete a state of mixture as the butter in milk when newly drawn from the cow.

It now remains with the physician to ascertain the therapeutic value of the formula. We hope that by its means our knowledge of the action of camphor as a remedial agent may become more full and precise than hitherto.—*Retrospect of the Medical Sciences.*

